# Long-Term Follow-Up of Patients with 46,XY Partial Gonadal Dysgenesis Reared as Males

**DOI:** 10.1155/2014/480724

**Published:** 2014-12-14

**Authors:** Juliana Gabriel Ribeiro de Andrade, Antonia Paula Marques-de-Faria, Helena Campos Fabbri, Maricilda Palandi de Mello, Gil Guerra-Júnior, Andréa Trevas Maciel-Guerra

**Affiliations:** ^1^Department of Medical Genetics, Faculty of Medical Sciences, State University of Campinas (UNICAMP), Rua Tessália Vieira de Camargo 126, 13083-887 Campinas, SP, Brazil; ^2^Interdisciplinary Group for the Study of Sex Determination and Differentiation (GIEDDS), State University of Campinas, Campinas, SP, Brazil; ^3^Center for Molecular Biology and Genetic Engineering (CBMEG), State University of Campinas, Campinas, SP, Brazil; ^4^Department of Pediatrics, Faculty of Medical Sciences, State University of Campinas, Campinas, SP, Brazil

## Abstract

*Background/Aims*. Studies on 46,XY partial gonadal dysgenesis (PGD) have focused on molecular, gonadal, genital, and hormone features; little is known about follow-up. Our aim was to analyze long-term outcomes of PGD. *Methods*. Retrospective longitudinal study conducted at a reference service in Brazil. Ten patients were first evaluated in the 1990s and followed up until the 2010s; follow-up ranged from 13.5 to 19.7 years. All were reared as males and had at least one scrotal testis; two bore *NR5A1* mutations. Main outcomes were: associated conditions, pubertal development, and growth. *Results*. All patients had normal motor development but three presented cognitive impairment; five had various associated conditions. At the end of the prepubertal period, FSH was high or high-normal in 3/6 patients; LH was normal in all. At the last evaluation, FSH was high or high-normal in 8/10; LH was high or high-normal in 5/10; testosterone was decreased in one. Final height in nine cases ranged from −1.57 to 0.80 SDS. All had spontaneous puberty; only one needed androgen therapy. *Conclusions*. There is good prognosis for growth and spontaneous pubertal development but not for fertility. Though additional studies are required, screening for learning disabilities is advisable.

## 1. Introduction

Partial gonadal dysgenesis (PGD), one of the 46,XY disorders of sex development (DSD) [1], is a rare disorder characterized by sex ambiguity due to variable degrees of testicular dysgenesis in individuals without a syndromic picture who have a normal male karyotype.

The histology of dysgenetic testes may vary from gonads with a few tubular structures and predominance of fibrous tissue to those with mild abnormalities, such as reduction of mean tubular diameter and mean number of germ cells and Sertoli cells per tubular profile [2]. Dysgenetic testes may be found bilaterally or may be associated with streak gonads, and the degree of embryonic Sertoli and Leydig cell dysfunction determines the degree of virilization of the internal and external genitalia [2]. Thus, the genital phenotype may range predominantly from male to female, including cases of marked sex ambiguity [3–5].

PGD was initially considered by many authors as a variant of 46,XY complete gonadal dysgenesis (CGD), which is characterized by bilateral streak gonads and female internal and external genitalia. However, mutations in* SRY* (*sex determining region Y*) gene, which have been described in many cases of XY CGD [6, 7], are rarely seen in PGD [8–10]. In recent years, both heterozygous and homozygous mutations in* NR5A1* (*Nuclear Receptor Subfamily 5, Group A, Member 1*) gene, which codifies the SF1 (*steroidogenic factor 1*) protein, have been found in about 15% of patients with PGD [11–13].

There is a high risk of germ cell neoplasia in the streak gonads of these individuals, which may reach 35% [1]; as a consequence, prophylactic gonadectomy is indicated [1, 14]. Tumors may also arise in the dysgenetic testes, particularly those with marked dysgenesis, which are not located in the scrotum [1, 15]. Thus, when the patients are raised as males, preservation of testes must be carefully evaluated.

The main differential diagnosis of PGD is mixed gonadal dysgenesis (MGD), one of the DSD associated with sex chromosome abnormalities [1]. PGD and MGD share similar gonadal and genital features; however, in MGD there is mosaicism with a 45,X cell line and one or more lineages with a normal or structurally abnormal Y chromosome [16]. As a consequence, patients with MGD may show clinical features of Turner syndrome, including short stature, dysmorphisms, and cardiovascular and renal malformations.

Distinguishing PGD from MGD depends on the karyotype, which must include the analysis of a sufficient number of cells to rule out mosaicism with high degree of confidence [17, 18].

Most studies on PGD focused on its gonadal and genital and sex hormone features and also on the search for mutations in genes involved in testis differentiation; however, little is known about other aspects of its clinical picture, including growth, puberty, and possible associated clinical conditions. As a consequence, when diagnosis is made, it is difficult to provide complete information to the parents on prognosis.

Between 1996 and 1998 we had the opportunity to evaluate 13 patients with PGD, all reared as males, using the same clinical and histopathological criteria [2]. These patients had also been subject to the same cytogenetic and molecular evaluations [2, 10], and many were followed in our University Hospital since then. The aim of this study was to analyze long-term follow-up of these patients, in order to better establish the prognosis of this condition.

## 2. Patients and Methods

Ten of 13 patients previously reported by our group (Scolfaro et al.) [2] were followed up in the University Hospital and were included in this study. Seven of them were regularly followed up in the pediatric endocrinology service and the other three were seen recently by us. These ten cases, described in Table 1, correspond to Scolfaro et al.'s cases 1, 3–9, 11, and 13.

The patients were first seen by us in the 1990s with ages ranging from 14 days to four years; nine were referred in the first year of life. In Scolfaro et al.'s study, the diagnosis of PGD was supported by the findings of ambiguous genitalia, a G-banded 46,XY karyotype with analysis of 16–32 cells, negative response of testosterone to hCG test without increase in precursors of testosterone synthesis (progesterone, dehydroepiandrosterone, and androstenedione), low AMH levels, presence of a streak gonad in two patients, and, in all cases, at least one gonad with histopathological features compatible with testicular dysgenesis (abnormal mean tubular diameter, severe tubular hypoplasia, low tubular fertility index, severe germinal hypoplasia, and/or hyperplasia of Sertoli cells).

At the time of the first visit to our service, assessment of luteinizing hormone (LH), follicle-stimulating hormone (FSH), progesterone, androstenedione, and dehydroepiandrosterone had been performed by radioimmunoassay in our service and anti-Müllerian hormone (AMH) by an enzyme-linked immunosorbent assay, using antibodies against human recombinant AMH in the laboratory of the Unité de Recherches sur l'Endocrinologie du Developpement (INSERM), Montrouge, France. The human chorionic gonadotropin (hCG) test had been performed with measurement of total testosterone levels before and 24 hours after the last of a series of 3 daily intramuscular injections of 2000 IU of hCG (Profasi, Serono) and was considered normal when the patient presented an increase in testosterone level of more than 4.9 nmol/L (1.4 ng/mL) [2].

Maternal age at birth ranged from 19 to 46 years (mean 28 years) and paternal age from 16 to 52 years (mean 31.1 years). Within nine full-term gestations, birth weight ranged from 2470 to 3750 g (mean 3097 g) and length from 46 to 51.5 cm, with an average of 48.4 cm. No patients had consanguineous parents and only two had a family history of genital ambiguity: case 2 (a first cousin once removed) and case 5 (maternal aunt and great-aunt).

The urethral meatus was most frequently penile (6/10). Bilateral dysgenetic testes were found in 6/10 cases and dysgenetic testis with contralateral streak was found in two. In the remaining cases there was unilateral dysgenetic testis; in one of the cases the contralateral gonad was absent and in the other it had not been biopsied. Müllerian derivatives were found in two patients. All patients were raised as boys and had at least one testis in the scrotum. All had absence of mutations in* SRY* or* WT1* (*Wilms Tumor 1*) genes [10]. Two of them (cases 2 and 3) had heterozygous* NR5A1* mutations, a p.Lys38^*^ and a p.Ser32Asn, respectively.

At the time of the last clinical evaluation in our service, patients' ages ranged from 15.5 to 19.8 years (mean 18 years); the mean time between first and last clinical evaluation in our service was 17.3 years (range 13.5–19.7 years). Follow-up data were obtained from the medical files and included neuromotor development, learning disabilities, congenital malformations, acquired diseases, concentrations of follicle-stimulating hormone (FSH), luteinizing hormone (LH), and total testosterone (determined by electrochemiluminescence), semen analysis, and occurrence of testicular neoplasia. The normal pubertal male range for FSH, LH, and testosterone was, respectively, 1.5–12.4 IU/L, 1.7–8.6 IU/L, and 2.86–8.10 ng/mL. Semen analysis was done according to the latest World Health Organization's guidelines [19].

Growth data were also collected from medical records, as well as data on pubertal development, which was evaluated considering the age of onset of the first signs of puberty and its progression. Patients' heights were expressed as standard deviation score (SDS) using reference data from CDC (NCHS/CDC 2000). Final height was considered when growth rate was ≤1.0 cm/year in patients who had completed pubertal development (at least Tanner stage 4). Whenever measurement of biological parents was available, the target height was calculated as (maternal height + paternal height + 12.5 cm)/2 ± 6,5 cm [20].

The Institutional Review Board approved this study (776/2007).

## 3. Results

All patients had normal motor development but three presented learning disabilities of unknown etiology (which were mild in two cases and moderate in one). Five had various associated conditions, including vesicoureteral reflux in two cases and facial dysmorphisms in one patient. Primary hypothyroidism with negative antithyroid antibodies was diagnosed in one of the patients with a* NR5A1* mutation when he was 12 years old (case 2); hearing loss due to middle ear infections, psychiatric problems, and obesity were also observed (one case each) (Table 2). There was no case of testicular neoplasia.

**Figure 1:**
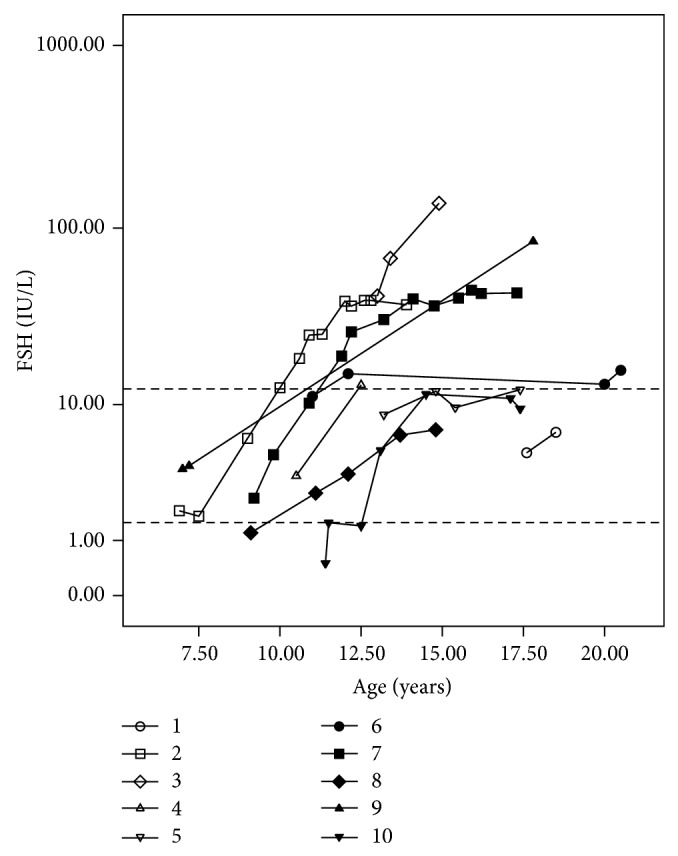
FSH levels measured by electrochemiluminescence at different ages in patients with partial gonadal dysgenesis. FSH values are presented on the *y*-axis on a logarithmic scale. Dotted lines on the *y*-axis represent the upper and lower normal limits for FSH levels in pubertal boys (1.5–12.4 IU/L).

**Figure 2:**
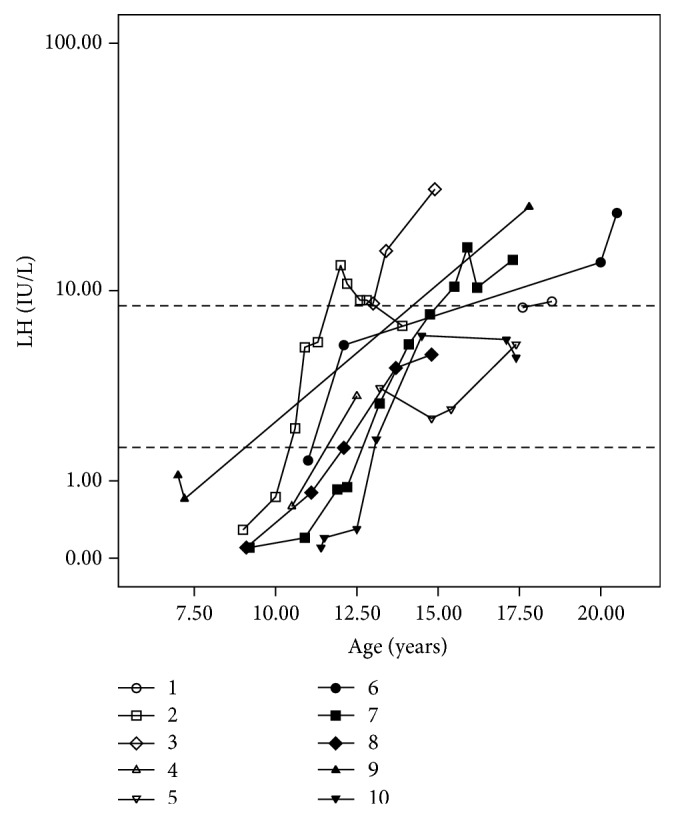
LH levels measured by electrochemiluminescence at different ages in patients with partial gonadal dysgenesis. FSH values are presented on the *y*-axis on a logarithmic scale. Dotted lines on the *y*-axis represent the upper and lower normal limits for LH levels in pubertal boys (1.7–8.6 IU/L).

Results of measurements of gonadotropins (FSH and LH) and testosterone during follow-up were also analyzed. Data on the measurement of gonadotropins at the end of the prepubertal period, between ten and 12 years, were available for six patients (cases 2, 4, 6, 7, 8, and 10). FSH levels were in the upper limit of the normal male pubertal range or higher in 3/6 patients (cases 2, 6, and 7), while LH levels were normal in all cases (Figures 1 and 2).

At the last hormone evaluation (mean 16.5 years; range 12.6–20.5 years) FSH concentrations were high in 4/10 patients (cases 2, 3, 7, and 9), in the upper limit in four (4, 5, 6, and 10), and normal in two (1 and 8) (Figure 1). In turn, LH concentrations were high in 4/10 patients (cases 3, 6, 7, and 9), in the upper limit in one (case 1), and normal in five (2, 4, 5, 8, and 10) (Figure 2).

**Figure 3:**
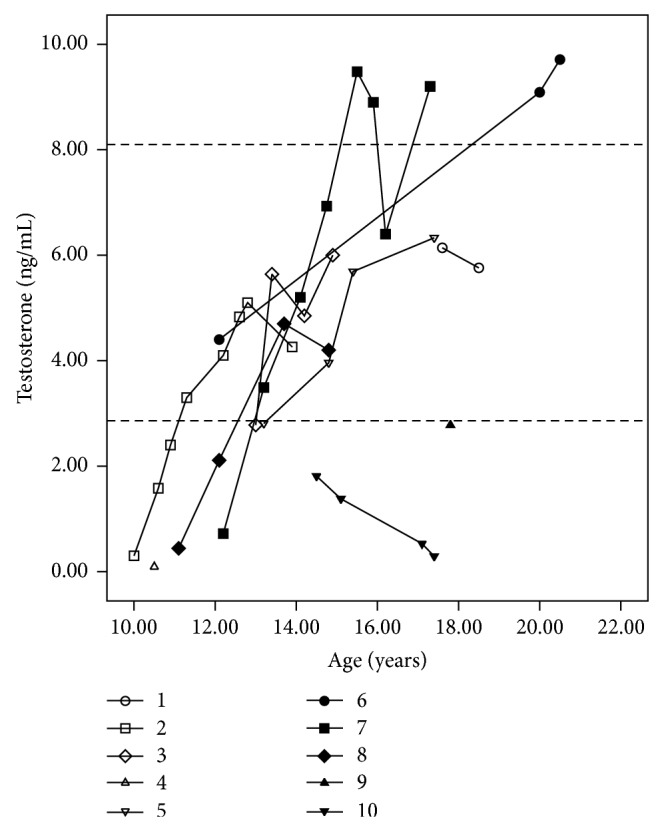
Testosterone levels measured by electrochemiluminescence at different ages in patients with partial gonadal dysgenesis. FSH values are presented on the *y*-axis on a linear scale. Dotted lines on the *y*-axis represent the upper and lower normal limits for testosterone levels in pubertal boys (2.86–8.10 ng/mL).

Measures of testosterone could be obtained from 9/10 patients in the pubertal age range. The levels remained normal in seven cases, remained at the low limit of normality in one, and decreased in one patient (case 10), who received androgen replacement therapy (Figure 3). Nine patients had reached final height, which ranged from −1.57 to 0.80 SDS, and in two of the eight cases for whom information on parental height was available it was lower than their genetically predicted range (target height ±6.5 cm). One patient (case 4) was still growing, with normal velocity and within the parental target range (Table 3).

Pubarche occurred at a mean age of 12.0 years (range 9–15 years). Eight patients had complete and spontaneous pubertal development (≥ Tanner stage 4), one was at stage G4P4 at 15.5 years (case 4), and another (case 10) received androgen therapy at 17.6 years, when pubertal development was G3P3; a few months later he reached stage G4P4. The seven patients who were regularly followed up in our pediatric endocrinology service had normal progression of puberty; most had low testicular volume. Three patients had a sperm count; all had severe oligozoospermia and low motility, and two had also abnormal semen viscosity.

## 4. Discussion

In DSD, sex assignment should be based on a precise diagnosis of the condition's underlying etiology. Together with genital appearance and surgical options, this will allow the establishment of a prognosis on the need for lifelong replacement therapy, potential fertility, and malignancy risk [1] and also possible associated conditions. In the case of PGD, however, prognosis is not yet clearly established.

Our results showed that all patients had normal neuromotor development, that most had normal growth, and that there was no consistent pattern of associated conditions. However, though learning disabilities are usually not a feature of DSD, in this sample it was observed in a significant proportion of cases (almost one-third), including a patient with moderate difficulties. Although this association may be casual, one may also consider the possibility that both conditions, testicular dysgenesis and cognitive impairment, have the same origin.

Interesting findings regarding our two patients bearing a* NR5A1* mutation are hypothyroidism in one of them and schizophrenia in the other. Acquired primary hypothyroidism has not been described as a feature of patients with PGD, with or without* NR5A1* mutations. The low prevalence of these conditions in young people aged 11–18 years (0.113%) and the fact that it is even rarer in males, with a 1 : 2.8 male to female ratio [21], make this finding noteworthy, even though expression of* NR5A1* mRNA in thyroid gland is very low [22]. On the other hand, there is a recent publication of two women with mutations in this gene who had psychiatric symptoms [23].

The absence of testicular tumors in this sample demonstrates that maintenance of testes in the labioscrotal folds of patients reared as males is a relatively safe procedure, at least until the end of puberty.

Relevant findings were obtained regarding pubertal development. Though positive results from hCG stimulation tests in infancy or childhood were obtained in 4/9 patients, spontaneous pubertal development occurred in all cases. Pubertal delay was not observed, and in 9/10 cases there was normal progression of puberty, which strongly indicates that there is a good prognosis regarding spontaneous puberty in PGD patients reared as males when at least one testis may be kept in the scrotum.

In fact, in 7/9 cases testosterone levels were in the normal range during adolescence, though a progressive rise of LH in half of the cases raises the possibility that Leydig cell dysfunction may become evident in adulthood. On the other hand, high levels of FSH in most patients, sometimes observed early in adolescence, indicated that reproductive function was impaired, which could be shown in those patients whose sperm count was obtained.

Some patients with* NR5A1* mutations and 46,XY PGD have been shown to have normal testosterone production in adolescence inducing spontaneous virilization [24–26], though follow-up indicated a progressive gonadal failure with elevated FSH in such cases. A similar picture was observed in our two patients with* NR5A1* mutations (cases 2 and 3) and also in the other eight cases without mutations in this gene. However, to the best of our knowledge, no other studies on long-term follow-up of patients with PGD reared as males are available to allow comparison with our sample.

## 5. Conclusions

Patients with PGD raised as males who have at least one testis in the labioscrotal region have a good prognosis for growth and spontaneous pubertal development but not for spontaneous fertility. Though additional studies are still required, our results also indicated that management of individuals with this condition should include screening for learning disabilities.

## Figures and Tables

**Table 1:** Description of the sample and data from the initial evaluation in our service.

Case	1	2	3	4	5	6	7	8	9	10

Age at last clinical evaluation (years)	18.4	18,3	17.7	15.5	17.4	19.8	17.5	18.6	18.5	17.9

Age at first visit (months)	2	6	3	0.5	7	1.5	48	6	0.5	3

Maternal age at birth	29	NA	38	19	23	46	19	27	32	19

Paternal age at birth	32	NA	38	36	23	52	16	32	28	23

Pregnancy complications	Hypertensive disorder	NA	—	—	Bleeding in the 1st trimester	Hypertensive disorder	—	Hypertensive disorder; preeclampsia	—	—

Birth weight (g)	3500	2800	3000	2470^*^	3000	3550	3750	1650^∗#^	2850	2950

Birth length (cm)	48	48	48	47	48	51.5	50	41	46	49

Family history of sex ambiguity	—	1st cousin once removed	—	—	Maternal aunt and great-aunt	—	—	—	—	—

Urethral meatus	PER	PEN	PER	PEN	PEN	PEN	NL	PEN	PER	PEN

Right gonad: type, location (age in months)	DT, SC (11)	Streak, IN (122)	DT, SC (36)	DT, SC (16)	DT, SC (16)	NB, SC (108)	DT, IN (84)	DT, IN (19)	DT, IN (26)	DT, IN (36)

Left gonad: type, location (age in months)	DT, SC (11)	DT, IN (122)	DT, SC (36)	DT, SC (16)	DT, SC (16)	DT, IN (108)	Absent	DT, SC (19)	Streak, AB (26)	DT, IN (36)

Internal genitalia	Normal male	UGS vagina and uterus	UGS blind-ending vagina	Normal male	Normal male	Normal male	Normal male	Normal	UGS rudimentary uterus	Normal male

Total testosterone (nmol/L) after hCG stimulation test (age in months)	1.4 (7)	<0.3 (44)	<0.3 (30)	1.7 (basal testosterone) (0.5)	<0.3 (10)	1.0 (92)	<0.3 (78)	<0.3 (13)	<0.3 (20)	<0.3 (31)

AMH (pmol/L) (age in months)^§^	118 (7)	71 (44)	52 (30)	98 (0.5)	114 (10)	113 (92)	73 (78)	107 (13)	11 (20)	25 (31)

AB: abdominal; DT: dysgenetic testis; IN: inguinal; NA: not available; NB: not biopsied (normal at palpation); NL: normal; PEN: penile; PER: perineal; SC: scrotal; UGS: urogenital sinus; AMH: anti-Müllerian hormone; mo: months.

^*^Small for gestational age; ^#^preterm gestation.

^§^Normal range: 0.5–12 mo = 251–679 pmol/L; 12.01–48 mo = 360–638 pmol/L; 48.01–84 mo = 309–566 pmol/L; 84.01–108 mo = 234–438 pmol/L.

**Table 2:** Neuromotor development and congenital and acquired diseases of ten patients with partial gonadal dysgenesis.

Case	1	2	3	4	5	6	7	8	9	10

Neuromotor development	Normal	Normal	Normal	Normal	Normal	Normal	Normal	Normal	Normal	Normal

Learning disabilities	Mild	—	—	—	—	Mild	—	—	Moderate	—

Associated conditions	Inguinal hernia		Club feet; right vesicoureteral reflux			Prominent antihelix; telecanthus; broad nasal bridge; high-arched palate		Small pseudodiverticulum of the urinary bladder; grade I left vesicoureteral reflux	Left renal cyst	

Other		Hypothyroidism obesity	Schizophrenia	—	—	Hearing loss due to middle ear infections	—		—	Dyserythropoiesis

**Table 3:** Data on growth, puberty, semen analysis, and surgical procedures undergone by ten patients with partial gonadal dysgenesis.

Patient	1	2	3	4	5	6	7	8	9	10

Target height (cm)	168.3	171.5	165.5	170	182.2	169.7	—	170.5	182.5	176.7

Target height (*z* score)	−1.17	−0.73	−1.57	−0.94	+0.77	−0.99	—	−0.87	+0.80	−0.0

Final height (cm)	170.2	175	165	∗	175	165.5	180	171	171	174

Final height (*z* score)	−0.89	−0.19	−1.51	—	−0.11	−1.58	+0.57	−0.76	−0.76	+0.30

Age of pubarche	12.5	9.5	13	12.5	11.5	15	11.5	10.5	11	13.5

Tanner staging at last visit (years)	G4P5 (18.4)	G5P5 (18.3)	G4P4 (17.7)	G4P4 (15.5)	G5P5 (17.4)	G5P5 (19.8)	G4P4 (17.5)	G4P4 (18.6)	G5P5 (18.5)	G3P3^#^ (17.6)/G4P4^§^ (17.9)

Testicular volume (mL) at last visit (right/left)	NA	4/8	2/5	6/8	12/12	20/—	20/—	10/20	NA	—/8

Sperm analysis (optical microscopy)	NP	NP	NP	NP	Low viscosity, rare spz (some motile)	High viscosity, rare spz (all immotile)	Normal viscosity, rare spz (some motile)	NP	NP	NP

Surgical procedures	Orthophalloplasty (11 mo); hypospadias correction (4 surgeries between 1 and 2 y)	Orchidopexy + hypospadias correction (2 y 8 mo)	Orchidopexy (3 y 3 mo); hypospadias correction (7 y); fistula correction (12, 13, 14, and 18 y)	Hypospadias correction (1 y 7 mo); fistula correction (2 y 8 mo and 2 y 9 mo)	Hypospadias correction (1 y 4 mo); fistula correction (4 y and 5 y)	Hypospadias correction (8 y 8 mo); fistula correction (9 and 12 y)	Orchidopexy (5 y)	Orchidopexy + orthophalloplasty (2 y 5 mo); fistula correction (3, 4, and 14 y)	Orchidopexy + hypospadias correction (2 y 2 m); fistula correction (5 and 9 y)	Inguinal hernia correction + orchidopexy (3 m); hypospadias correction (4 y)

^*^Did not attain final height; ^#^before testosterone replacement and associated with gynecomastia; ^§^after testosterone replacement; NA: not available; NP: not performed; spz: spermatozoa.
